# Three-Dimensional Printing of Rigid–Flexible Ceramic–Epoxy Composites with Excellent Mechanical Properties

**DOI:** 10.3390/ma18071479

**Published:** 2025-03-26

**Authors:** Zhaozhi Wang, Biao Jiang, Yajie Liu, Zhiheng Xin, Zhibin Jiao

**Affiliations:** School of Mechanical Engineering, Shenyang University of Technology, Shenyang 110870, China; zhaozhi_wang@sut.edu.cn (Z.W.); 13364513802@163.com (B.J.); ddliuyajie@163.com (Y.L.); 16688375411@163.com (Z.X.)

**Keywords:** 3D printing, biomimetic gradient structure, rigid–flexible composites, ceramic–epoxy dual-phase composites, flexural strength, fracture toughness

## Abstract

Inspired by the Bouligand structure of the mantis shrimp’s dactyl club, in this study, we employed direct ink writing 3D printing technology to fabricate bioinspired gradient ceramic samples with varying gradient spacings and rotation angles. A rigid–flexible coupled bioinspired gradient ceramic–epoxy resin composite was successfully constructed based on epoxy resin infiltration. The effects of gradient variations and rotation angles on mechanical properties were systematically investigated with flexural strength and fracture toughness tests. The experimental results revealed that, at a fixed rotation angle, both the flexural strength and fracture toughness initially increased and then decreased with an increase in gradient spacing. The infiltration of epoxy resin significantly enhanced the mechanical performance of the composite samples. Specifically, the maximum flexural strength of 63.35 MPa was achieved at Δd = 0.08 and a rotation angle of 12°, while the highest fracture toughness of 2 MPa/m^2^ was observed at Δd = 0.1 and a rotation angle of 12°. A failure analysis indicated that the introduction of gradient structures and epoxy resin infiltration altered the failure forms of traditional ceramics, with the primary toughening mechanisms including crack deflection, fiber pull-out, and crack branching. In this study, we successfully developed a rigid–flexible coupled bioinspired gradient ceramic–epoxy resin composite with excellent mechanical properties based on bioinspired design and gradient optimization, providing new insights and methodologies for the design and fabrication of high-performance ceramic materials.

## 1. Introduction

With the rapid progress of modern science and industrial technology, the application of structural materials in the field of engineering is becoming more and more extensive in scope, along with an increase in the comprehensive performance requirements of materials [1,2,3]. Therefore, as a class of typical materials with high-temperature stability and excellent mechanical properties [4,5], ceramic materials have gradually assumed an important position in various diversified fields, such as the military [6], aerospace [7], the electrical industry [8,9], automotives, and aircraft [10,11]. However, despite their promising applications, traditional ceramic materials, constrained by the bonding mode of intermolecular chemical bonds and complex crystal structures, exhibit inherent characteristics of high brittleness and low fracture toughness, which have become key factors limiting their widespread use under extreme or complex working conditions. Therefore, exploring and optimizing the conflicting relationship between strength and toughness in ceramic materials have emerged as core issues urgently needing resolution in the field of ceramic materials research.

**Figure 1:**
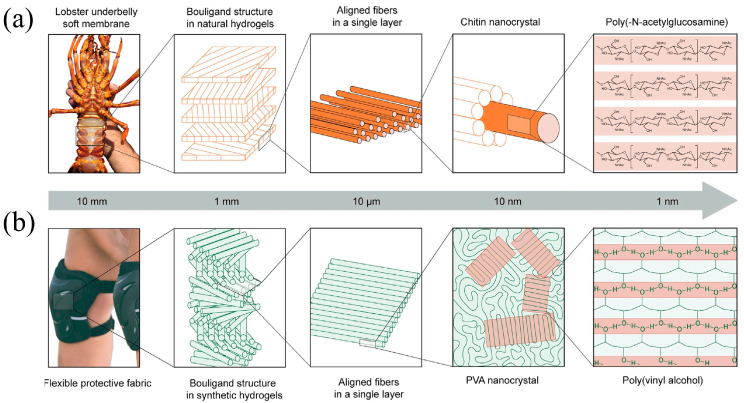
The bioinspired design of strong and fatigue-resistant nanofibrous hydrogels. (**a**) The natural hydrogel membrane in a lobster underbelly is composed of the Bouligand-type multilayered structure. (**b**) Synthetic nanofibrous hydrogels can potentially be used as flexible protective fabric materials [23].

During hundreds of millions of years of biological evolution and natural selection, various organisms in nature have evolved different self-protection mechanisms in order to cope with changing competitive environments and enhance their ability to survive. In this process, many organisms have naturally developed biostructural composites with complicated structural designs. These materials are characterized by the synergistic action of multiple structural elements within a single organism, which not only strengthens the overall structure but also enhances its toughness while maintaining a lighter mass and displaying excellent mechanical properties [5,12,13]. Typical examples of natural biomimetic materials include bamboo, horns, shellfish, crustaceans, and human and animal bones and teeth. These natural biomaterials generally have multiple levels and multiscale structural characteristics, allowing for a good balance between structure and performance and endowing them with light weight [13], high strength, high toughness, and excellent impact resistance [14,15,16]. Based on the observation of multiple structures of various organisms, researchers have found that the Bouligand structure, which consists of the periodic helical arrangement of uniaxial nanofibers, is a typical strategy found in nature. The Bouligand structure has superior energy absorption capacity under external loading by rotating and reorienting the ordered nanofibers [17]. It is widely found in the bodies of various stomatopods [18], such as the bulbous rods of mantis shrimps [19], arapaima gigas scales [20], crab exoskeletons, and scorpion claws [21,22]. Examples of studies on this type of structure include that by Zhao et al., who prepared a nanofiber hydrogel with excellent strength properties and fatigue resistance by mimicking the Bouligand structure of the lobster abdomen (Figure 1) [23], and Pinho et al., who designed and fabricated Bouligand-type biomimetic laminate samples by using ultrathin carbon fiber prepregs and carried out full-penetration quasi-static indentation tests, which showed that the Bouligand structure has higher load-bearing capacity and energy absorption capacity than conventional structures [24]. In addition, biomimetic composites with Bouligand structures were able to achieve crack deflection in three dimensions, thus delaying catastrophic failure more effectively.

In addition, the ‘brick and mortar structure’ in the pearl layer of shells is another representative natural biomaterial that has attracted extensive attention among researchers. In the pearl layer, well-arranged inorganic nanoscale calcium carbonate nanoplates act as ‘bricks’, while *β*-chitin and silk proteins act as ‘mortar’; the former are filled with organic chitin and silk proteins to form a ‘brick–mortar’ structure with good mechanical properties [25,26]. Such a composite structure further enhances the toughness of the composite material due to the filling of organic phases.

However, as natural biological structures are very intricate, it is difficult to fabricate ceramic composites with complex biomimetic structures with traditional processing methods. Therefore, finding an advanced processing method is the focus of research on the preparation of biomimetic structures. Among known techniques, 3D printing, an emerging manufacturing method, allows for the precise control and generation of ceramic scaffolds due to its layer-by-layer fabrication and digital control characteristics [27,28]; in particular, the 3D printing process helps to accurately control the microstructure of the material, and the fine filamentary structure thus obtained can force cracks to deflect on different scales, thus optimizing the fracture toughness of the material. This method thus represents a new avenue for the preparation of bionic complex structural ceramics.

Based on the above research ideas, a large number of researchers have used 3D printing and infiltration technology to prepare a series of rigid–flexible coupled bionic composite ceramics with excellent mechanical properties based on the bionic concept of combining soft and hard characteristics. Among the materials used, rigid ones refer to ceramics with a certain degree of strength, while flexible materials are generally polymers, including resin and polyurea. The purpose of such selection is to combine the high strength of ceramics and the toughness of polymers [29,30,31]. Specifically, for ceramic–resin composites, ceramic acts as a strong phase to improve the strength of the composites, while resin acts as a soft phase to optimize their toughness by hindering crack extension. Therefore, as it combines rigid biomimetic ceramic structures with flexible resin materials, the construction of rigid–flexible coupled biomimetic ceramic–resin composites is an effective method for improving fracture toughness and expanding the application scope [32,33,34].

There have been several studies on this topic. For instance, Gan et al. [35] successfully printed alumina ceramic lattices with different topologies with stereo-lithography 3D printing and prepared composites with two continuous component materials interpenetrating each other based on resin infiltration, and the results showed that the synergistic effect between tough epoxy resin and strong ceramic lattices in interpenetrating phase composites (IPCs) can significantly improve mechanical strength and energy absorption and maintain good mechanical strength. Zhai et al. [36] successfully used a combination of DLP 3D printing and polymer infiltration technologies to prepare ceramic–epoxy hierarchical interpenetrating phase composites (HIPCs) with excellent mechanical properties.

Although existing research has successfully developed biomimetic ceramic composites with excellent mechanical properties, the inherent low toughness of traditional ceramic materials has not yet been fundamentally improved. At the same time, current research lacks in-depth and thorough investigations into the toughening mechanism of bionic ceramic composite materials at the microscopic level and especially the systematic analysis of the influence of the bionic design of the variables (such as angle, spacing, and the proportion of each component) on the overall mechanical properties and the microscopic toughening mechanism. Therefore, the interactions between these complex variables and their specific contributions to overall structural toughness enhancement need to be further investigated.

## 2. Materials and Methods

### 2.1. Raw Materials

Alumina powder acted as the main body of the slurry, which determined the performance of the slurry. The selected raw material was alumina powder (*α*-Al_2_O_3_, purity > 99.24%, d_50_ = 0.7–1.3 μm, Anhui Estone Material Technology Co., Ltd., Hefei, China). Silicon dioxide powder (SiO_2_, d_50_ = 100 nm, Suzhou Beesley New Materials Co., Ltd., Suzhou, China) was used as a sintering aid. Sodium hexametaphosphate ((NaPO_3_)_6_, SHMP, Tianjin Hengxing Chemical Preparation Co., Ltd., Tianjin, China) was used as a dispersant. Polyvinylpyrrolidone (PVP K15, Hefei BASF Bio-technology Co., Ltd., Hefei, China) was used as a stabilizer. Sodium alginate (SA, Shanghai RHAWN Chemical Technology Co., Ltd., Shanghai, China) was used as a thickener. Poly (ethylene glycol) (PEG 6000, Shanghai Aladdin Biochemical Technology Co., Ltd., Shanghai, China) was used as a lubricant. Deionized water was prepared in the laboratory and was used as a solvent.

### 2.2. Slurry Preparation for 3D Printing

**Figure 2:**
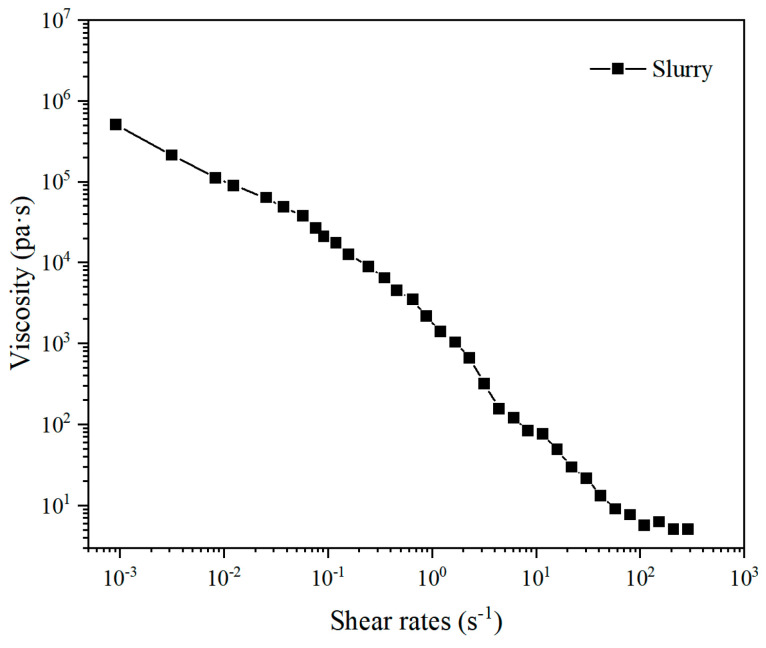
Shear rheology properties of ceramic ink.

Al_2_O_3_ powder was used as the main phase and SiO_2_ powder as the secondary phase. The overall mass of the two was taken as the mass of the powder, and the mass ratio of the main and secondary phases was set to 13:1. The detailed composition of all other materials is listed in Table 1. (NaPO_3_)_6_ and K15 both accounted for 3 wt% of the total weight of the powder, PEG 6000 for 1 wt%, and SA for 0.36 wt%. A total of 9 g of deionized water was used as the solvent. First, alumina and silica particles were fully dried in a drying oven to ensure the accuracy of the weighing process. Subsequently, (NaPO_3_)_6_ and SiO_2_ were added to deionized water and then stirred at a speed of 600 rpm for 10 min to obtain a silica suspension. The latter was uniformly mixed with K15, PEG 6000, and SA and then stirred at a speed of 600 rpm for 10 min. Finally, alumina particles were added to prepare ink in several rounds, after each of which stirring was performed at a speed of 800 rpm for 10 min. All mixing was performed by utilizing a planetary ball mill. The solid-phase content of the slurry was calculated as 71 wt%. The rheological test results of the slurry were presented in Figure 2.

### 2.3. Direct Ink Writing 3D Printing of Gradient Bouligand Structure

**Figure 3:**
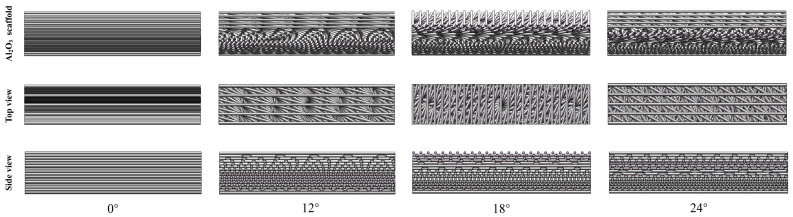
Schematic structure of biomimetic gradient ceramics with different deflection angles at same gradient spacing.

The 3D printing equipment used in this study was a Bio-Architect^®^SR-type (Hangzhou Regenovo Biotechnology Co., Ltd., Hangzhou, China) 3D printer. According to the actual printing setup and in order to make the printed structure more in line with the bionic structure design, the gradient spacings were selected as Δd = 0, Δd = 0.08, Δd = 0.1, and Δd = 0.12. Moreover, in order to maintain consistency with the arrangement of the organism’s own helical pattern and balance the thickness of the sample and the integrity of the helical period, for the test, we selected three angles of 12°, 18°, and 24° close to a representative range of rotation angles (0–20°) of common biological Bouligand structures found in nature [17,37,38]. Upon rotation through a certain number of layers, the three types of angles formed a specific periodic arrangement. The specific model structure is shown in Figure 3. For the convenience of the subsequent presentation, we uniformly denoted by solid ceramics the plain Bouligand ceramic specimens without gradient changes, while the remaining groups were named by using the nomenclature of Al_2_O_3_/EP-Δd-deflection angle. It is worth noting that the model structure designed in this study was only a theoretical model based on the consideration of the actual printing process and each parameter fit. There may be a small gap between the theoretical model and the printed samples, and the latter were subjected to specific tests. To this end, the number of layers of the printed samples was set to 16 layers, and we adjusted the other printing parameters as follows: each printing layer had a height of 0.5 mm, the printer nozzle diameter was 0.6 mm, the control nozzle pressure was 0.1 Mpa, and the nozzle moving speed was 11 mm/s. Each printing layer was completed by changing the print pitch and deflection angle to achieve the gradient Bouligand structure. We started printing after setting each print parameter.

### 2.4. Debinding and Sintering

**Figure 4:**
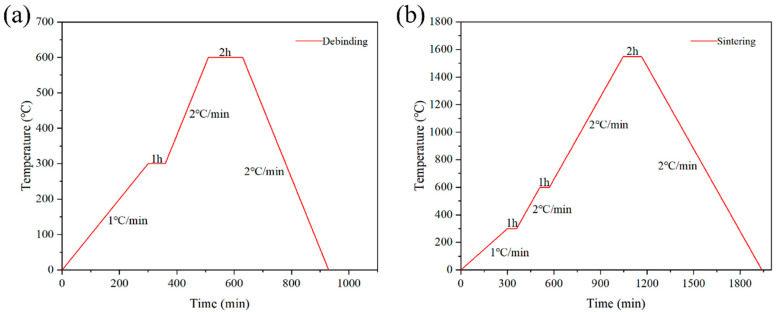
Heat treatment curve of alumina ceramics: (**a**) debinding curve, (**b**) sintering curve.

First, the printed samples were placed at room temperature for 24 h to dry them completely. Afterwards, their surface was sanded until smooth for subsequent heat treatment and resin penetration. We then placed each sample in a debinding furnace (FX2CT-60/11, Hefei Facerom Thermal Energy Equipment Co., Ltd., Hefei, China) and increased the temperature to 300 °C at a constant heating rate of 1 °C/min. The temperature was held at 300 °C for 1 h; then, it was further increased to 600 °C at a heating rate of 2 °C/min and held for 2 h. The purpose of this step was to remove excess organic matter from the alumina ceramic blanks and initially improve the mechanical strength of the debinding parts by performing pre-sintering at a temperature of 600 °C. It is worth noting that the sample must be heated up at a slow rate during the debinding process; otherwise, performance defects such as the bending, slumping, and deformation of the ceramic part will occur, affecting the overall appearance and mechanical properties of the sample. The debinded samples were cooled to room temperature and transferred to a sintering furnace (FMJ-12/18, Hefei Facerom Thermal Energy Equipment Co., Ltd., Hefei, China) for sintering. The temperature was first increased from room temperature to 300 °C at a heating rate of 1 °C/min and held for 1 h, then to 600 °C at a heating rate of 2 °C/min and held for 1 h, and finally to 1550 °C at a heating rate of 2 °C/min and held for 2 h. Finally, the alumina ceramic specimens were obtained by cooling the samples to room temperature at a cooling rate of 2 °C/min after the completion of sintering. The specific debinding and sintering curves are shown in Figure 4.

### 2.5. Fabrication of Bioinspired Composites

The ceramic–epoxy composite materials were prepared by infiltrating resin into the sintered ceramic scaffolds. The epoxy resin and curing agent (315-AB, Wenzhou Ying Partner Technology Co., Ltd., Wenzhou, China) were proportioned according to a mass ratio of 3:1. Firstly, we weighed a certain mass of epoxy resin and poured it into a container; then, we added the curing agent proportionally and used a glass rod for constant stirring. The mixed epoxy resin was placed in a vacuum-drying oven until all the bubbles it contained disappeared. Next, the samples to be infiltrated were placed in pre-designed molds, where the epoxy resin was then slowly and evenly poured. The epoxy resin was fully infiltrated into the ceramic samples to close the distance between them and the gradient Bouligand samples and fix any defects formed in the printing process, thus obtaining a dense ceramic–epoxy composite structure. Finally, the successfully infiltrated samples were placed in a room-temperature environment for 24 h to allow the epoxy resin to be fully cured.

### 2.6. Structural Characterization

The macroscopic and microstructures of the biomimetic gradient sample were observed using optical microscopy. Field emission scanning electron microscopy, FESEM (ZEISS GeminiSEM 300, Jena, Germany), was used to characterize the microstructure of Al_2_O_3_ ceramics’ cross-section of the sample, and the elemental distribution of the sample section was tested and analyzed using an SEM piggybacked energy spectrometer. Before SEM, the samples were bisected and gold-coated to observe the cross-section. X-ray optical microscopes (ZEISS Xradia 620 Versa, Jena, Germany) were utilized to observe the microscopic 3D morphology of the fractured samples as well as the crack paths. The rheological behavior of the slurry at room temperature was characterized by a rotating rheometer (NETZECH Kinexus prime pro+, Selb, Germany). The diameter of the rheometer was 25 mm.

### 2.7. Mechanical Testing

In this study, three-point bending and fracture toughness tests were performed on the sintered ceramic specimens by using a universal testing machine (model UTM 6000, Shenzhen Suns Technology Stock Co., Ltd., Shenzhen, China). In order to comply with test-related standards, the dimensions of the flexural strength specimens were approximately 50 × 8 × 8 mm^3^, with a spanning distance of 40 mm and a constant loading rate of 0.5 mm/min. For the fracture tests, the dimensions of the specimens were approximately 30 × 8 × 8 mm^3^, with a spanning distance of 24 mm and a constant loading rate of 0.05 mm/min. Unlike the three-point bending tests, the fracture toughness tests required the implementation of the Single-Edge Notched Beam (SENB) method. For SENB tests, it is necessary to prefabricate a crack at the bottom center of the specimens, where the requirements of the size of the crack are as follows: its notch width needs to be no greater than 0.25 mm, and the notch depth should be about 1/4–3/4 of the height of the specimen. Therefore, the specimen was notched in the bottom center section with a diamond saw to a depth of about 2 mm and a width of about 0.2 mm, and at least three samples were tested for each set of parameters to obtain statistically reliable values. In order to calculate the flexural strength and fracture toughness of the sample, the flexural strength (*σ*) was calculated with the equation below:

(1) $$\sigma =\frac{3FL}{2bh^{2}}$$

In this equation, *σ* is the flexural strength, *F* is the break force in the test, *L* is the outer (support) span, *b* is the specimen width, and *h* is the specimen height.

The fracture toughness *K_IC_* was calculated by the following equation:

(2) $$K_{IC}=Y\frac{3FL}{2bh^{2}}\sqrt{a}$$

In this equation, *K_IC_* is the fracture toughness, *F* is the maximum force, *L* is the outer (support) span, *b* is the side-to-side dimension of the test specimen perpendicular to the crack length, *h* is the top-to-bottom dimension of the test specimen parallel to the crack length, *a* is the crack depth, and *Y* is the stress intensity factor coefficient, determined by the following equation:

(3) $$Y=1.93-3.07\frac{a}{h}+14.53{\left(\frac{a}{h}\right)}^{2}-25.11{\left(\frac{a}{h}\right)}^{3}+25.8{\left(\frac{a}{h}\right)}^{4}$$

During the fracture test, considering the absorbed energy per unit area, the work of fracture (*W_f_*) was calculated by the following equation:

(4) $$W_{f}=\frac{A}{2b\left(h-a\right)}$$

In this equation, *A* is the area under the load–displacement curve, which represents the energy of the applied external load during the fracture test, and *W_f_* is the work of fracture.

**Figure 5:**
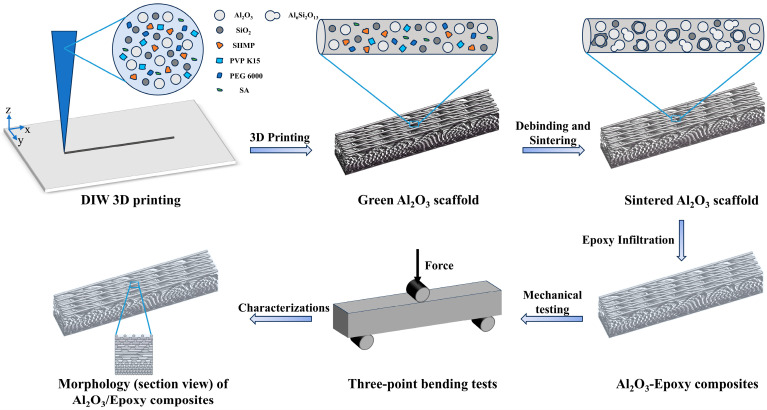
Schematic illustration of preparation process of rigid–flexible coupled biomimetic gradient ceramic–epoxy composites.

The schematic diagram of the specific preparation process was shown in Figure 5.

## 3. Results and Discussion

### 3.1. Morphologies of Al_2_O_3_ Scaffolds and Al_2_O_3_–Epoxy Composite Structures

**Figure 6:**
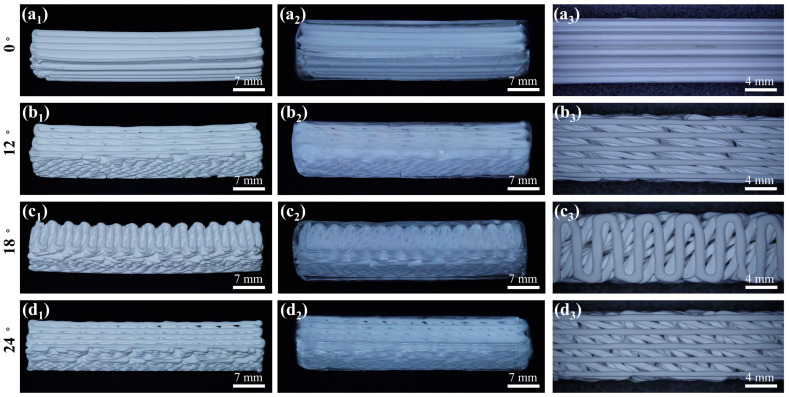
Macroscopic and microscopic morphologies of biomimetic gradient ceramic scaffold and ceramic–epoxy composites with different deflection angles for Δd = 0.08: (**a1**–**a3**) 0°, (**b1**–**b3**) 12°, (**c1**–**c3**) 18°, and (**d1**–**d3**) 24°.

Figure 6(a1–d1) show the overall appearance of the biomimetic gradient ceramic structure after heat treatment. Macroscopically, the printed gradient Bouligand structure was not significantly different from the designed 3D model, and all the samples maintained relatively good integrity, which indicates that the 3D printing technology has certain advantages in the fabrication of complex ceramic structures. Further, the microstructure shown in Figure 6(a3–d3) indicates a clear layer-by-layer rotational feature in the printing direction. As a result, high printing accuracy can be achieved with the direct writing 3D printing process. After heat treatment, all samples underwent some degree of sintering shrinkage of about 5%, while no additional cracks or flaws were detected in the sintered body, thus implying that the gradient Bouligand structure obtained after preliminary printing and sintering already possessed a certain degree of mechanical properties in its own right. Additionally, the macroscopic morphology of the samples after epoxy infiltration is shown in Figure 6(a2–d2). From the external view, the ceramic and epoxy phases are interpenetrated and tightly integrated. From the above macroscopic and microscopic morphology features, it can be seen that Al_2_O_3_ ceramic scaffolds with complex shapes can be prepared by additive manufacturing and then combined with the resin infiltration process to obtain Al_2_O_3_–epoxy composite structures. The results show that it is feasible to prepare ceramic–resin composite structures with excellent strength and toughness with this method.

### 3.2. Mechanical Properties

**Figure 7:**
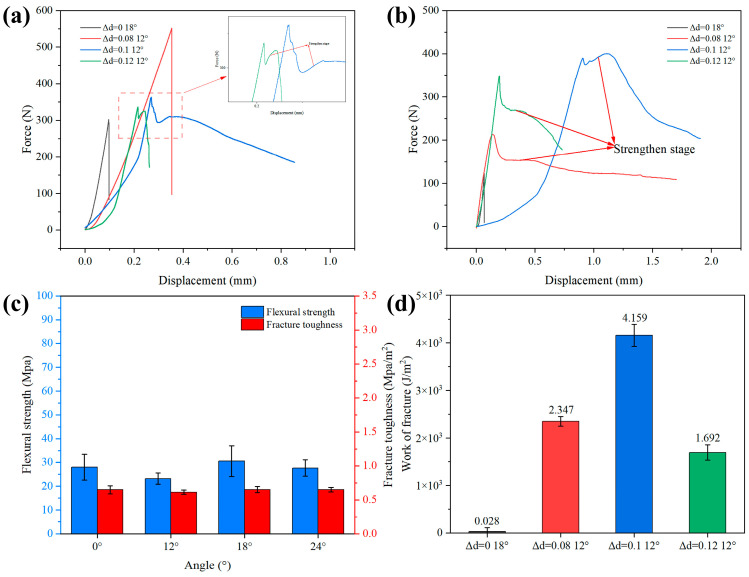
Load–displacement curves of rigid–flexible coupled biomimetic gradient ceramic–epoxy composites with relatively good mechanical properties with different gradient increments. (**a**) Flexural strength specimen. (**b**) Fracture toughness specimen. (**c**) Statistics of mechanical properties of solid ceramics without silica. (**d**) Work of fracture.

In order to better illustrate the role of silica, in the subsequent discussion, before focusing on the overall mechanical properties of the bionic gradient composite ceramics, we first analyze the mechanical properties of the sintered ceramics without the addition of silica. As shown in Figure 7c, the flexural strength and fracture toughness of the solid ceramics without the addition of silica were slightly decreased compared with those of the solid ceramics to which silica had been added, indicating that the introduction of silica had a certain degree of positive influence on the solid ceramics’ mechanical properties, though the enhancement effect was relatively insignificant. Specific reasons will be discussed later.

Figure 7 shows the load–displacement curves of the samples with relatively high flexural strength and fracture toughness for different gradient spacings. It can be seen that the ceramic–epoxy composites, due to their structural properties and the presence of resin, show a typical strengthening stage in the load–displacement curves of some samples, featuring a more gradual and progressive mechanical behavior. In contrast, the two groups of flexural strength samples, Al_2_O_3_/EP-0-18° and Al_2_O_3_/EP-0.08-12°, both showed brittle fracture modes. After reaching the yield point, the peak force dropped rapidly, and catastrophic failure occurred. The potential reason for the brittle fracture of the flexural samples of the Al_2_O_3_/EP-0.08-12° group could be that in the specific printing process, due to the limited preparation process, the ideal precise control of the spacing of the bottom layer is often difficult to achieve. This limitation leads to the gradient spacing of the bottom layer being too narrow, which in turn affects the ability of the epoxy resin to infiltrate sufficiently and uniformly into the various layers and spacing of the sample. Due to the insufficient penetration of the epoxy resin, the bottom of the sample maintained the original state of ceramics on the macroscale but failed to form the expected composite structure; thus, its mechanical properties were affected. As a result, when subjected to external forces, the sample exhibited brittle fracture characteristics similar to those of a single-ceramic material, and its failure form was no different from that of an ordinary Bouligand sample. Among the fracture toughness samples, all groups of gradient Bouligand samples showed a typical strengthening stage. Moreover, the work of fracture of the bionic gradient composite ceramics was obtained by calculating the load–displacement curves of the three-point bending test. As shown in Figure 7d, in each group of samples, the work of fracture reached the maximum value of 4.159 KJ/m^2^ when Δd = 0.1 and the deflection angle was 12°, which was much higher than that of solid ceramics, indicating that the bionic gradient composite ceramics consume more loading energy during the fracture process under this condition. As reported in a related study [39], the resin component plays a key role in subjecting ceramic–resin composites to external loads. Its unique presence allows the composite to act as a vital slip system, effectively transferring the internal stress from the ceramic layer to the resin matrix. This slip process is usually accompanied by longer displacements, thus preventing catastrophic failure in the composites. The ceramic–resin composites showed higher flexural strength and fracture toughness than traditional ceramic. Moreover, the load–displacement curves of the two groups of fracture toughness samples with Al_2_O_3_/EP-0.1-12° and Al_2_O_3_/EP-0.12-12° both showed a step-like fracture mode. It is worth mentioning that this step-like fracture mode is usually found in ceramic–metal or ceramic–polymer composites and has been proven to play an important role in preventing instant catastrophic failure in engineering materials [40].

**Figure 8:**
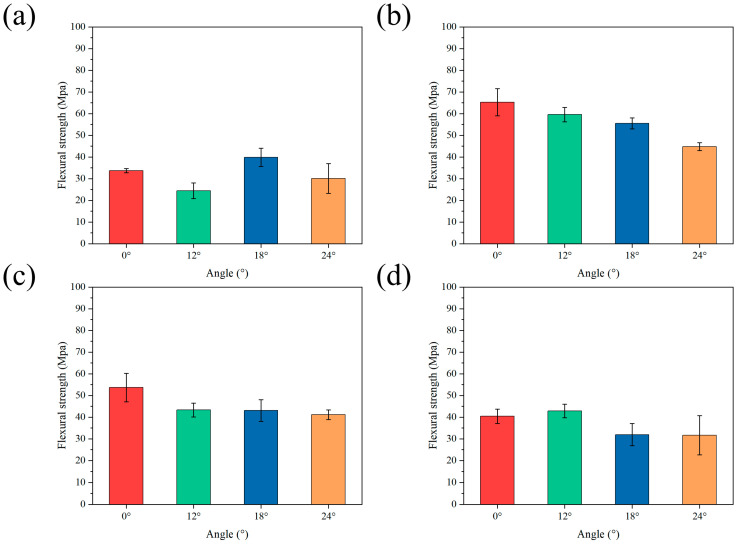
Flexural strength statistics of biomimetic rigid–flexible coupled gradient ceramic–epoxy composites with different gradient increments: (**a**) Δd = 0, (**b**) Δd = 0.08, (**c**) Δd = 0.1, (**d**) Δd = 0.12.

**Figure 9:**
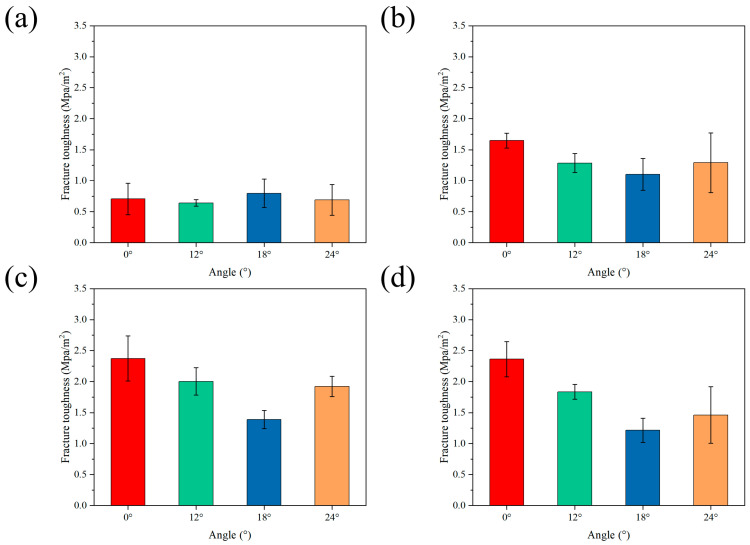
Fracture toughness statistics of biomimetic rigid–flexible coupled gradient ceramic–epoxy composites with different gradient increments: (**a**) Δd = 0, (**b**) Δd = 0.08, (**c**) Δd = 0.1, (**d**) Δd = 0.12.

The mechanical properties of the Al_2_O_3_–epoxy gradient ceramics were tested. The sintered structures with different gradient modes were tested for flexural and fracture properties, and the curves obtained are shown in Figure 8. In terms of flexural strength, it can be seen from Figure 8a that in the ordinary Bouligand samples without epoxy infiltration, when Δd = 0, the flexural strength tends to decrease and then increase with an increase in the angle of deflection; on the contrary, when the angle of deflection continues to increase, the flexural strength of the solid ceramic samples decreases. Regarding fracture toughness (see Figure 9), when Δd = 0, its trend in the solid ceramic samples is the same as that of flexural strength. In the gradient Bouligand samples with gradient spacing, after resin infiltration, the flexural strength decreases gradually with an increase in the deflection angle for both Δd = 0.08 and Δd = 0.1. When Δd increases to 0.12, the ceramic bracket and epoxy resin act synergistically to jointly transfer the load. Moreover, the presence of two phases results in the redistribution of stress within the material when the composite structure is subjected to loads, and cracks are also deflected by the interpenetrating structure formed by the two phases, which results in an increase in flexural performance [41,42,43]. However, the enhancement effect was relatively limited, and there was only a small increase when the deflection angle was 12°, while the flexural strength decreased when the deflection angle continued to increase.

The statistical analysis of the fracture toughness curves indicates that the three kinds of fracture samples with gradient spacing exhibit similar mechanical behavior characteristics within the deflection angle range of 0–18° (Figure 9b,c). Within this range, as the deflection angle increases, the fracture toughness exhibits a monotonic decreasing trend. When the deflection angle continues to increase, the fracture toughness demonstrates a notable upward trend, with a more pronounced increase than the flexural strength. However, compared with the samples with a single-gradient structure (without deflection angle), the gradient samples with deflection angles exhibit a slight decline in the overall mechanical properties. This phenomenon can be attributed to the lack of an effective synergistic effect between the gradient spacing and the deflection angle at smaller deflection angles, leading to a decrease in fracture toughness within the region of low deflection angles.

As can be seen from the above, the flexural strength and fracture toughness of the samples corresponding to each gradient spacing experienced a period of decline, but compared with the ordinary Bouligand structure without epoxy infiltration, there was a significant increase in the mechanical properties. For example, as shown in Figure 8, when Δd = 0.08, the flexural strength corresponding to the various angles had values of 65.23 Mpa, 59.57 Mpa, 55.49 Mpa, and 44.75 Mpa, showing increases of 93.3%, 143.8%, 39.3%, and 48.7%, respectively, compared with solid ceramic. Moreover, when Δd = 0.08 and the deflection angle was 12°, the flexural strength of the gradient Bouligand sample reached a maximum value of 59.57 MPa. Regarding fracture toughness, as shown in Figure 9, when Δd = 0.1, the fracture toughness corresponding to each angle had values of 2.37 Mpa/m^2^, 2 Mpa/m^2^, 1.39 Mpa/m^2^, and 1.92 Mpa/m^2^, showing increases of 238.5%, 212.5%, 73.8%, and 178.3%, respectively, compared with solid ceramic. Finally, the fracture toughness of the gradient Bouligand sample reached a maximum value of 2 MPa/m^2^ when Δd = 0.1 and the deflection angle was 12°.

In summary, the gradient Bouligand structure not only possesses inherent reinforcing properties that allow a significant increase in strength to be achieved but can also effectively regulate the spatial distribution of the components in the composite material and optimize its microstructure. The Bouligand structure shows significant superiority in terms of the effectiveness of fracture toughness enhancement. Further, the peak force performance of gradient Bouligand composites can be further enhanced by introducing the gradient design concept to construct biomimetic gradient composites with rigid–flexible coupled properties. This finding not only contributes to composite design research but also provides an important theoretical basis and practical guidance for the preparation and application of high-performance composites.

### 3.3. Microstructure Analysis

**Figure 10:**
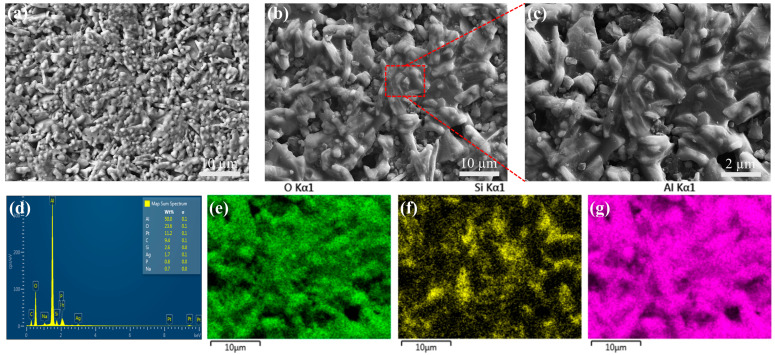
Microstructure comparison of sintered ceramics with and without silica and microstructure and elemental distributions of biomimetic gradient ceramics: (**a**) without silica, (**b**,**c**) with silica, (**d**) EDS curve, and (**e**–**g**) distribution of constituent elements.

In this study, a detailed microstructural analysis of the alumina-based sintered ceramics with added silica was carried out by using scanning electron microscopy (SEM). In order to better illustrate the role of silica, a control group was set up as a sintered ceramic without added silica. Specific observations are shown in Figure 10, where it can be seen that the addition of appropriate levels of silica promotes the growth of alumina grains to a certain extent.

The results in Figure 10a–c show that the addition of silica significantly promoted the formation of a liquid phase among the alumina grains, mainly surrounding them and forming an extensive liquid-phase region. The presence of this liquid phase positively affected the sintering process by strengthening the bonding between the grains and facilitating the migration of grain boundaries, thus significantly enhancing the completeness and densification of the sintering process. Moreover, under high-temperature sintering conditions, silica reacts chemically with alumina to generate a mullite phase consisting of [SiO_4_] and [AlO_4_] tetrahedral structural units. The formation of mullite enhances the strength of the sintered ceramics to a certain extent, which is due to the high mechanical strength and thermal stability of mullite itself. However, the presence of periodic oxygen vacancies in the mullite structure leads to the formation of a large number of holes and porous structures, which reduces the overall densification of the sintered ceramic [44]. In addition, it is worth noting that mullite produces cracks due to volume shrinkage at certain temperatures. These cracks not only destroy the integrity of the ceramic material but also reduce its mechanical properties. Therefore, although the formation of mullite contributes to strength enhancement in sintered ceramics, its negative effects should not be overlooked.

In order to verify the above observations, the elemental distribution of the sintered ceramic specimens was determined in this study by using the energy-dispersive spectroscopy (EDS) technique. As shown in Figure 10d–g, the EDS patterns clearly demonstrate the enrichment of Al and O elements in the alumina particles, while Si elements are uniformly dispersed in the alumina matrix. This result reconfirms the presence of liquid-phase silica in the sintered ceramics and corroborates the SEM observations.

**Figure 11:**
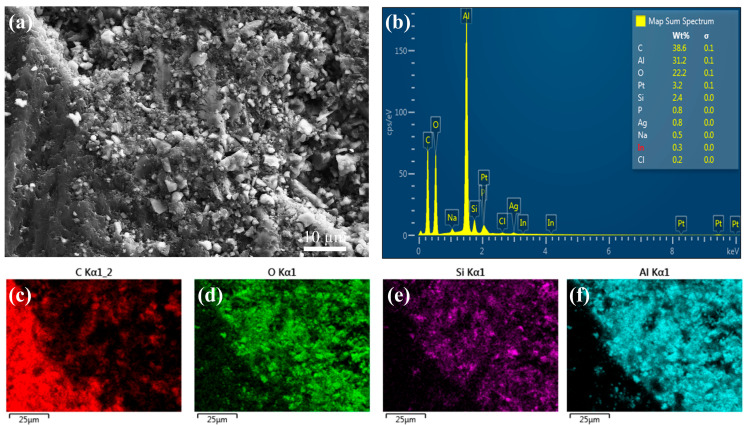
Microstructure and elemental distributions of rigid–flexible coupled biomimetic gradient ceramic–epoxy composites: (**a**) SEM image, (**b**) EDS curve, and (**c**–**f**) distribution of constituent elements.

Furthermore, we explored the microstructure of the biomimetic gradient Al_2_O_3_/EP composites after penetration by epoxy resin in depth. As shown in Figure 11a, it was observed that the interfacial bonding between the alumina particles and the epoxy resin was tight and intact, with no obvious interfacial defects or separation. Further elemental analyses showed that the Al and O elements were mainly concentrated inside the ceramic filaments. It is worth noting that both alumina and epoxy resin possess hydroxyl (-OH) surface functional groups. These functional groups can form hydrogen bonds under appropriate conditions, thus further enhancing the interfacial bonding between the alumina particles and the epoxy resin. The formation of hydrogen bonds not only improved the interfacial stability of the composites but also helped to improve the overall mechanical properties and durability of the composites [30,36,45].

### 3.4. Analysis of Toughening Mechanisms

It has been shown that the mechanical behavior of the Bouligand helical laminated structure, especially its load-bearing capacity and energy absorption properties, exhibits a significant dependence on the interlayer helix angle [46,47]. Specifically, as the helix angle changes, the distribution and magnitude of stresses within the monolayers and at the interfaces with the neighboring layers change significantly. These changes not only directly affect the load carrying capacity of the single-layer structure and its damage mode but also play a decisive role in the load transfer efficiency in the multilayer structure. In addition, the change in the helix angle between the layers triggers a damage mechanism whereby cracks progressively expand in a spiraling fashion between neighboring layers, a mechanism that further affects the overall mechanical properties of the material.

**Figure 12:**
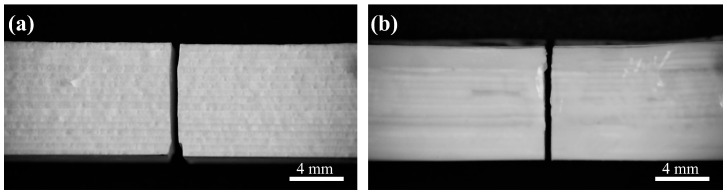
Fracture morphology of solid ceramic and gradient ceramic samples without deflection angle: (**a**) solid ceramic and (**b**) gradient ceramic.

For normal samples with neither gradient spacing nor deflection angles, cracks were preferentially generated where the bond was weak, and specimens with this structure failed immediately upon cracking, with little or no load-bearing capacity, so that the bond between the paths was less strong than the paths themselves (Figure 12a). In contrast, the deflection of the crack, as well as minor transverse expansion, could be clearly seen in the specimens with a certain deflection angle of the biomimetic Bouligand structure, which indicates that crack deflection occurs in three-dimensional space, effectively increasing the absorption of crack diffusion energy during the fracture process, thus enabling the specimen to withstand a larger ultimate load. At the same time, too large a deflection angle guides crack deflection poorly, and the crack continues to spread in the direction of the load without sufficient torsion, so the specimen absorbs less energy during the fracture process and can withstand a small ultimate load.

Some related studies have shown that in the ideal state, where only the crack de-flection mechanism exists, the larger the deflection angle, the larger the surface area of the fracture surface caused by crack deflection, the higher the energy required to form the deflected fracture surface, and the larger the energy dissipation. More specifically, the higher the external load required for increasing crack deflection, the higher the fracture toughness of the sample; however, in the specific experimental process of the present study, the maximum value was reached at a deflection angle of 18° in the case of solid ceramic specimens, and as the deflection angle continued to increase, the fracture toughness decreased instead. This was due to the existence of other toughening mechanisms, and when the crack was deflected to a certain degree, the path itself was not conducive to its continued expansion, resulting in the sudden failure of the sample.

**Figure 13:**
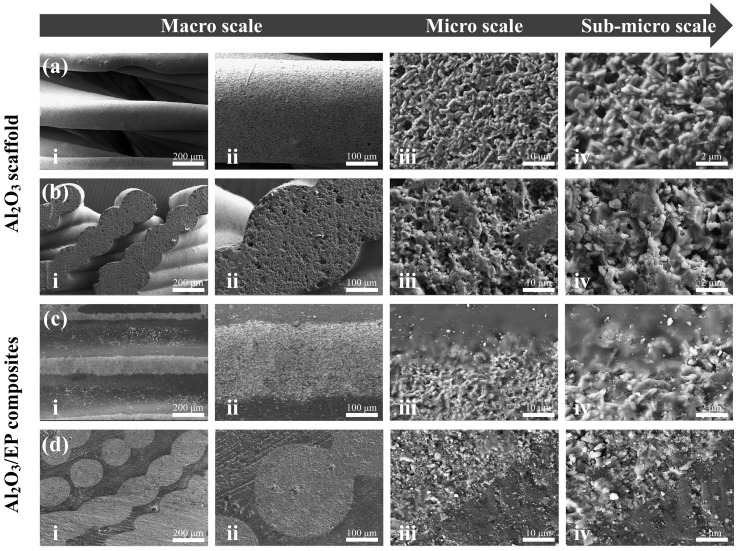
SEM images of solid ceramics and epoxy-infiltrated ceramic scaffolds. (**a**) The top surface and (**b**) cross-section of solid ceramic scaffolds. (**c**) The top surface and (**d**) cross-section of epoxy-infiltrated ceramic scaffolds.

Among the ceramic–epoxy composites, according to the in-depth analysis of the fine hierarchical structure shown in Figure 13, the superior mechanical properties and excellent energy absorption characteristics of the high-strength composites stem from the effective combination of the synergistic strengthening and toughening mechanisms of the internal multi-components. At the macro- and microscale levels, high-performance composite materials with sophisticated geometric design enable the ceramic scaffold to play a crucial role when subjected to loads. These scaffold structures not only significantly enhance the overall mechanical properties of composite materials but also endow them with superior strength characteristics. This reinforcing effect enables the composite material to maintain a stable structural form when subjected to external loads, effectively resisting deformation and damage, thus guaranteeing the reliability and durability of the material under extreme conditions.

At the same time, the gradient structure in the ceramic scaffold provided sufficient deformation space for epoxy resin. When subjected to external forces, the epoxy resin was able to undergo plastic deformation and absorb and disperse mechanical energy through this process. This plastic deformation mechanism not only effectively reduces the stress concentration phenomenon of the material but also converts mechanical energy into other forms of energy through energy conversion, achieving the efficient absorption and dispersion of energy. This multiscale synergistic strengthening and toughening effect endows high-strength composites with excellent mechanical properties and attractive energy absorption characteristics when subjected to external loads.

**Figure 14:**
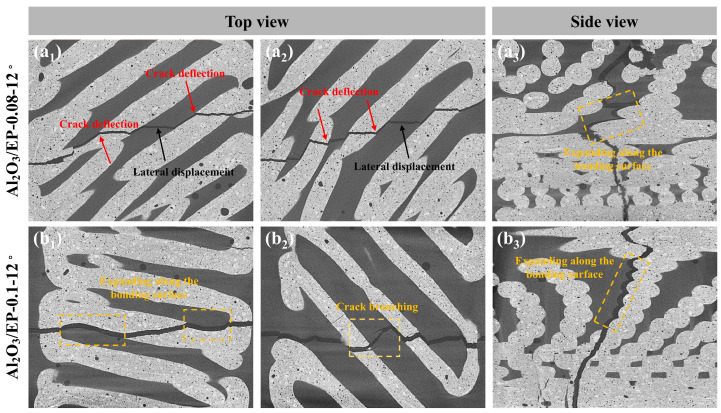
Micro-CT images of ceramic–epoxy composites after fracture with different gradient increments. (**a1**,**a2**) Top view of Al2O3/EP-0.08-12°, (**a3**) Side view of Al2O3/EP-0.08-12°. (**b1**,**b2**) Top view of Al2O3/EP-0.1-12°, (**b3**) Side view of Al2O3/EP-0.1-12°.

Figure 14 shows the final fracture morphology of solid ceramic specimens and ceramic–epoxy composite specimens subjected to quasi-static loading. For the flexural strength and fracture toughness samples with a deflection angle of 0°, the crack extension paths of all specimens were relatively regular, regardless of whether there is a gradient spacing variation or not. When the gradient spacing was fixed at Δd = 0.08, the degree of crack torsion of the composite specimens in three-dimensional space was gradually weakened with a change in the deflection angle compared with solid ceramics, which can be attributed to the basic failure mechanism similar to that of solid ceramics. Although the introduction of epoxy resin partially absorbed the mechanical energy through its plastic deformation ability, it did not fundamentally change the crack expansion pattern at larger deflection angles. However, different from the solid samples, it can be additionally observed from the two-dimensional cross-section of the sample that the cracks expand along the bonding area between the ceramic and the epoxy resin (Figure 14(a1–a3)), because in the fracture behavior of laminated helical composites, the crack will tend to avoid the hard matrix with high strength when expanding between layers and choose to expand along the interface where the bonding is weaker, as well as in the process of expansion, due to the existence of angular difference between the layers, which makes the crack deflect to different degrees when passing through the various layers. With the continued increase in the load, the crack expansion path begins to transfer to the epoxy resin component, accompanied by a significant lateral displacement process. In this process, the epoxy resin plays a large role, absorbing most of the energy and effectively inhibiting the further expansion of the crack so as to achieve the toughening effect. On the other hand, when the deflection angle was maintained at a certain value and the gradient spacing was increased to 0.12, a decreasing trend both in flexural strength and fracture toughness was observed. And at the same time, it was observed that a penetrating fracture crack is not the only one that exists inside the sample, but crack branching is also found in the adjacent parts of the penetrating crack (Figure 14(b2)). This is because the increase in gradient spacing indirectly reflects the increase in the content of epoxy resin. Specifically, the content of epoxy resin shows an increasing trend with increasing spacing. Compared with solid ceramics, ceramic–epoxy composites with gradient spacing design show significant superiority in terms of mechanical properties. However, an unlimited increase in the epoxy resin content cannot continuously improve the mechanical properties of composites. When the content reaches a certain threshold, its further growth will be accompanied by a significant increase in interfacial defects, which, as a potential stress concentration area, are prone to becoming a stress concentration point when subjected to external stresses, leading to the destruction of the material at a lower level of stress, which will weaken the mechanical properties of the composites and limit the maximum load-bearing capacity of the composite. In view of this, the present study emphasizes that the relationship between the gradient spacing and the epoxy resin content must be finely tuned when optimizing the preparation process of ceramic–epoxy composites in order to explore and achieve the optimal equilibrium between the two, aiming at maximizing the mechanical properties of the composites while avoiding interfacial strength degradation and the problem of premature failure due to the excessively high epoxy resin content. This finding provided an important theoretical basis and practical guidance for the design and development of high-performance composites.

**Figure 15:**
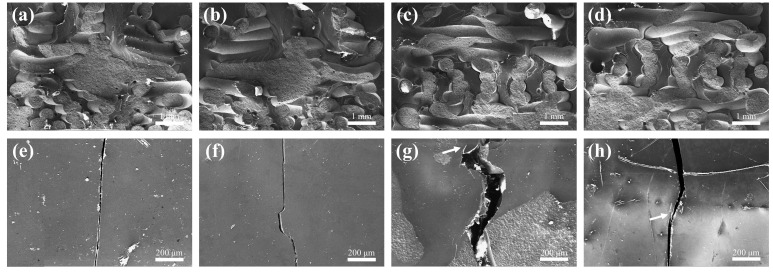
When Δd = 0.1, individual fracture samples showed ceramic filament pull-out and crack branching phenomena: (**a**,**b**) 12°, (**c**,**d**) 24°, and (**e**–**h**) SEM images of rigid–flexible coupled bionic gradient ceramic–epoxy fracture samples at different deflection angles (angle of deflection from left to right was 0°, 12°, 18°, and 24°, and white arrows show crack branching).

Among the fracture samples, in the 0–18° interval, the fracture toughness of the three groups of bionic gradient samples decreases continuously with the increase in the deflection angle, for reasons consistent with the previous analysis of the flexural strength curves. However, different from flexural strength, the fracture toughness of each group of samples rises when the deflection angle increases to a certain degree, indicating that an additional toughening mechanism was triggered at this angle. Upon closer inspection, it was found that at this angle, tiny ceramic filaments could be seen pulling out of the cross-section, and there was crack branching in some samples (see Figure 15h), both of which led to more energy dissipation during the test.

Another form of failure was the occurrence of interfacial debonding with the combination of the ceramic matrix and epoxy resin. Interfacial debonding refers to the phenomenon whereby the interface between two different parts of a material (such as fibers and matrix) loses its bonding force when subjected to an external force, with the two parts thus separating or sliding relatively to each other (see Figure 15a–d). There are two main ways to assess interfacial debonding: One is based on the maximum shear stress criterion, i.e., interfacial debonding occurs when the interfacial shear stress of a composite material under load reaches its interfacial shear strength. The other one is based on the critical energy release rate criterion of fracture mechanics, where the debonding region of the interface is regarded as an interfacial crack, and the debonding of the interface mainly depends on the interfacial energy balance associated with the fracture energy. In composites, interfacial debonding generally starts at the matrix crack, and debonding occurs at the interface near the matrix crack when the size of the latter is large enough and the external load reaches a critical value. Therefore, the toughening mechanism of the composites was not of a single origin but the result of the combined effect of several mechanisms.

## 4. Conclusions

In summary, in this study, inspired by the unique Bouligand structure found in nature, a bionic gradient ceramic–epoxy biphasic composite was successfully prepared by combining the gradient design concept with advanced 3D printing and infiltration techniques. In order to evaluate its mechanical properties, we performed quasi-static loading tests, and the results showed that the introduction of epoxy resin had a significant reinforcing effect on the bionic gradient ceramic structure. Further analyses of the main fracture morphology and toughening mechanisms of the composites show that the excellent mechanical properties of the bionic composites can be mainly attributed to the intricate hierarchical structural design of the gradient Bouligand structure, as well as the excellent rigid–flexible coupled matching mode, which corresponded to a variety of toughening enhancement mechanisms, including but not limited to crack deflection, interfacial debonding, and fiber pull-out. These toughening mechanisms effectively dissipate more fracture energy and prevent brittle fracture behavior by acting synergistically at multiple scales. Therefore, this study provides a practical way to prepare new composites with high performance and complex structures. In addition, the methods and techniques used in this study are widely applicable and can be generalized to the exploration and practical application of various material combinations.

## Figures and Tables

**Table 1:** Compositions of Al_2_O_3_ ceramic slurry.

**Ceramic Powder**	**Sintering Aid**	**Dispersant**	**Stabilizer**	**Lubricant**	**Thickener**	**Solvent**
*α*-Al_2_O_3_ (g)	SiO_2_ (g)	(NaPO_3_)_6_ (wt%)	K15 (wt%)	PEG 6000 (wt%)	SA (wt%)	Deionized water (g)
13	1	3	3	1	0.36	9

## Data Availability

The original contributions presented in this study are included in the article. Further inquiries can be directed to the corresponding author.
